# Transneuronal Degeneration in the Brain During Glaucoma

**DOI:** 10.3389/fnagi.2021.643685

**Published:** 2021-04-06

**Authors:** Mengling You, Rong Rong, Zhou Zeng, Xiaobo Xia, Dan Ji

**Affiliations:** ^1^Department of Ophthalmology, Xiangya Hospital, Central South University, Changsha, China; ^2^Hunan Key Laboratory of Ophthalmology, Changsha, China

**Keywords:** brain, glaucoma, neurons, neurodegenerative disease, RGC

## Abstract

The death of retinal ganglion cells (RGCs) is a key factor in the pathophysiology of all types of glaucoma, but the mechanism of pathogenesis of glaucoma remains unclear. RGCs are a group of central nervous system (CNS) neurons whose soma are in the inner retina. The axons of RGCs form the optic nerve and converge at the optic chiasma; from there, they project to the visual cortex *via* the lateral geniculate nucleus (LGN). In recent years, there has been increasing interest in the dysfunction and death of CNS and retinal neurons caused by transneuronal degeneration of RGCs, and the view that glaucoma is a widespread neurodegenerative disease involving CNS damage appears more and more frequently in the literature. In this review, we summarize the current knowledge of LGN and visual cortex neuron damage in glaucoma and possible mechanisms behind the damage. This review presents an updated and expanded view of neuronal damage in glaucoma, and reveals new and potential targets for neuroprotection and treatment.

## Introduction

**Figure 1:**
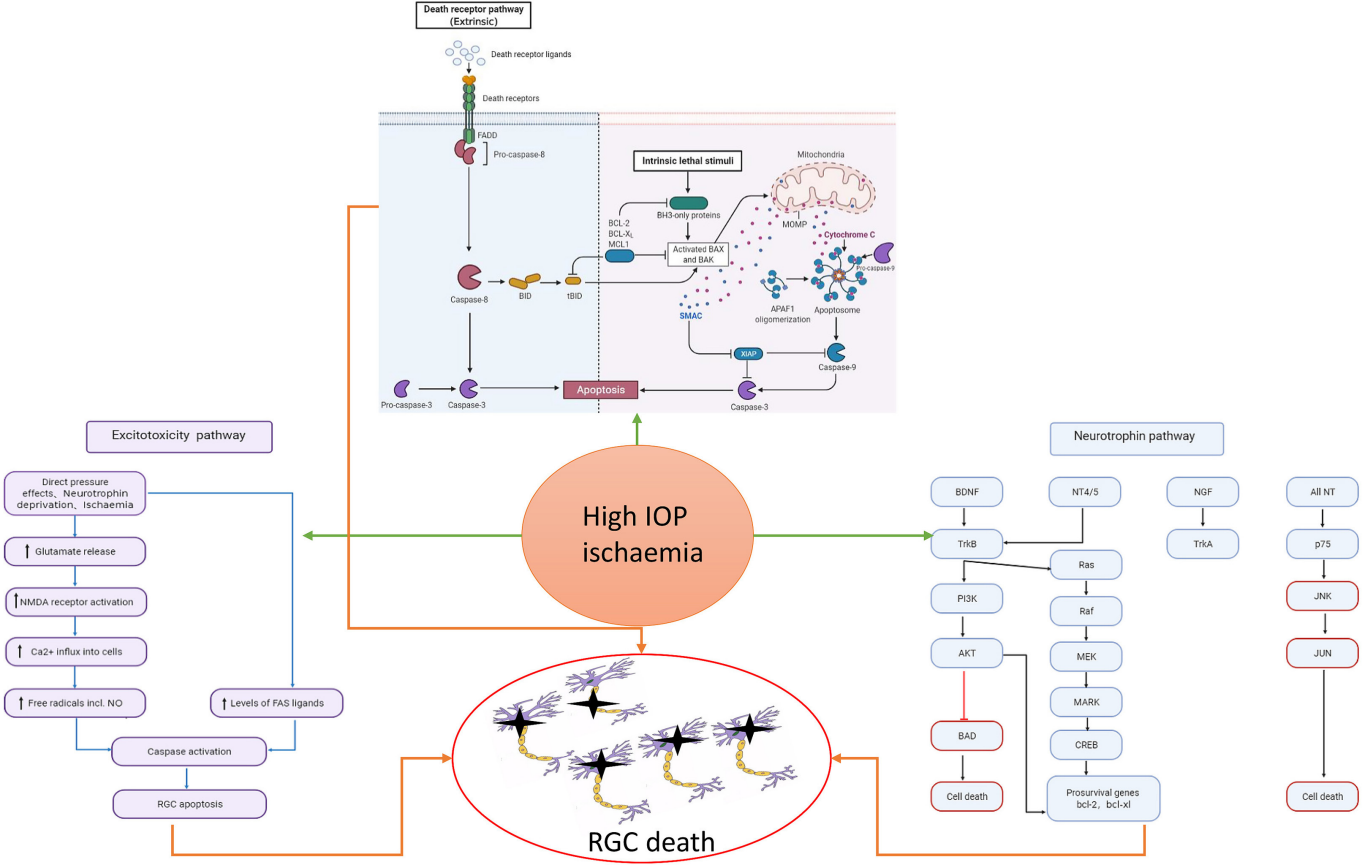
Risk factors such as high intraocular pressure and ischemia can cause the activation of cell apoptosis, the increase of excitotoxicity, and the imbalance of neurotrophic pathway, which will eventually cause the death of RGC. In the neurotrophic pathway, blue represents the pathway through which neurotrophic factors function under normal conditions, and red represents the pathway through which RGC damage is caused by stimulation.

Glaucoma, a group of diseases categorized by characteristic optic nerve damage and loss of visual field, is currently the most common cause of irreversible blindness in the world. It is estimated that there will be 111.8 million glaucoma patients worldwide by 2040 (Bourne et al., 2013; Tham et al., 2014; Jonas et al., 2017). Although the pathophysiological mechanisms behind glaucoma are not fully understood, it is acknowledged that high intraocular pressure is one of the most important risk factors for the onset of glaucoma (Sommer, 1989; Kass et al., 2002; Ma et al., 2019; Kim et al., 2020). High intraocular pressure can cause mechanical axonal damage and interrupt nutrient transmission, eventually causing apoptosis of retinal ganglion cells (RGCs) and loss of vision (see Figure 1) (Almasieh et al., 2012). As a result, the most predominant current treatment method for glaucoma is to decrease intraocular pressure (Fechtner and Weinreb, 1994; Burgoyne et al., 2005; Weinreb et al., 2014). However, some patients with normal intraocular pressure glaucoma, or undergoing intraocular pressure reduction, still experience further optic nerve damage and vision loss (Levene, 1980; Heijl et al., 2009; Musch et al., 2009; Garcia et al., 2019). The specific mechanism behind this phenomenon remains unclear.

Studies have shown that axon degeneration precedes RGCs apoptosis in glaucoma models (Crish et al., 2010). Furthermore, recent studies have demonstrated that astrocyte and macrophage-mediated demyelination precedes the loss of axons at cellular level (You et al., 2019). Glaucoma was once thought to be an axonal disease (Calkins and Horner, 2012). Clinical studies on glaucoma patients have shown that the distal axon is one of the first areas to disappear in chronic glaucoma (Sponsel et al., 2014). Several studies have found that there is great similarity between the pathology of glaucoma and that of central nervous system degenerative diseases (Ghiso et al., 2013; Jones-Odeh and Hammond, 2015; Mirzaei et al., 2017; Artero-Castro et al., 2020; Masri et al., 2020). Due to advances in neuro-imaging technology, increasing attention is being paid to changes in the brain visual pathways of patients with glaucoma (Danesh-Meyer and Levin, 2015; Kasi et al., 2019; Trivedi et al., 2019). Recently, increasing evidence shows that neuronal damage in glaucoma spreads *via* transneuronal degeneration, in which primary neuronal damage has a far-reaching effect on synaptic-connected distant neurons, a process known as transsynaptic degeneration (Weber et al., 2000; Lei et al., 2008; Hendrickson et al., 2015; Lawlor et al., 2018; You et al., 2019). This process may involve both anterograde and retrograde transneuronal degeneration (see Figure 3).

Previous studies have shown that in glaucoma models, apoptosis of RGCs can lead to optic nerve and optic tract atrophy, and further lead to degeneration and atrophy of the lateral geniculate nucleus (LGN), optic radiation, and visual cortex through transneuronal degeneration (Vrabec and Levin, 2007; Jindahra et al., 2009; Chen et al., 2013a; Rossiter, 2015). Sasaoka et al. used a primate monkey model to show that chronic ocular hypertension can significantly reduce the density of RGCs and LGN neurons in retina as well as light sensitivity of visual field (Sasaoka et al., 2008). Imaging studies have shown that there are changes in blood flow in the visual cortex of patients with normal-tension glaucoma and primary open-angle glaucoma (POAG) (Li et al., 2020; Wang et al., 2021). Concurrently, a large number of studies have shown that there are other types of neuronal damage in retina in addition to RGC damage (Agudo-Barriuso et al., 2013; Vidal-Sanz et al., 2015). Some researchers believe that RGC transneuronal degeneration leads to degeneration and atrophy of retinal bipolar cells (BC) and photoreceptors, resulting in damage to visual function and vision loss (Calkins, 2012). Based on this accumulated evidence, we believe that primary glaucoma is not only a simple ophthalmopathy, but also a systemic disease involving the central nervous system (CNS) through transneuronal degeneration. Optic nerve injury in primary open-angle glaucoma is a pathological factor which is not only unique to RGC apoptosis caused by pathological intraocular hypertension but is also related to LGN, optic radiation, visual cortex injury, and RGC apoptosis.

This paper summarizes the injury of brain neurons in glaucoma, focusing on injury of LGN and visual cortex, and also the mechanism behind this injury.

## Transneuronal Degeneration in Glaucoma Brain

**Figure 2:**
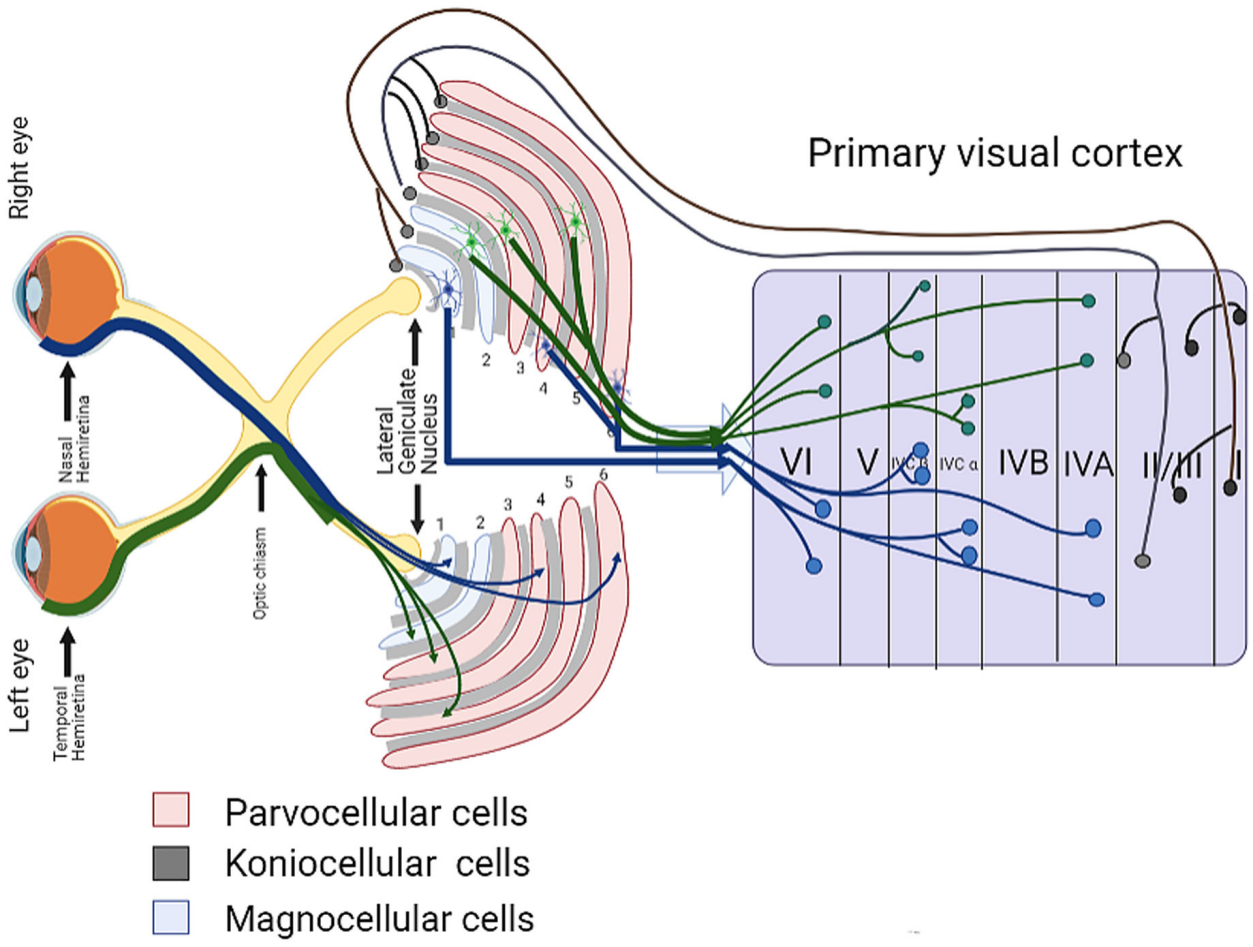
Schematic diagram of visual pathway, lateral geniculate body and visual cortex.

**Figure 3:**
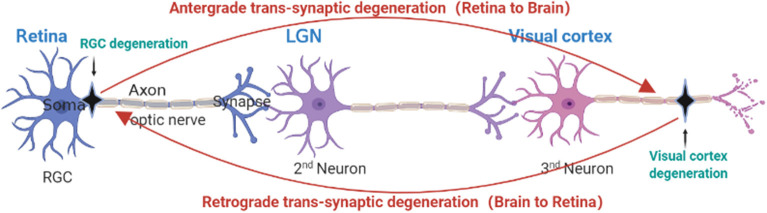
Anatomical depiction of retrograde and anterograde transsynaptic degeneration in the visual pathway. Retrograde trans-synaptic degeneration describes the process by which damage to the posterior visual pathway (black) results in subsequent retinal degeneration. Anterograde trans-synaptic degeneration describes the process by which retinal degeneration leads to subsequent degeneration of the posterior visual pathway.

Primary neuronal injury affects distal neurons, a process known as transsynaptic or transneuronal degeneration (Michelson et al., 2011). Transneuronal degeneration is known to be related to the pathological transmission of CNS degenerative diseases (Lopresti, 2018). In recent years, a large number of studies have shown that pathological damage in glaucoma is also spread in this way. Transneuronal degeneration can be divided into anterograde and retrograde depending on the site of origin and direction of spread (Kratchmarov et al., 2012). RGC injury has been proven to be the main pathological feature of glaucoma, and RGC damage can lead to optic nerve and optic tract atrophy, which in turn leads to LGN, optic nerve radiation, and visual cortex degeneration and atrophy through anterograde transneuronal degeneration (Vernazza et al., 2020). Figure 2 shows the complete visual pathway, LGN and visual cortex, and Figure 3 shows the transneuronal degeneration process after RGC injury. This process has been confirmed in primate glaucoma models and in glaucoma patients.

### The Degeneration of LGN in Glaucoma

After optic nerve formation, the RGC axons of humans and primates converge at the optic chiasma and then form optic tracts, and about 90% of RGC axons eventually terminate in the LGN (Yucel, 2013). In the visual system of mice, the axons of RGCs project to the subcortical pathway, mainly the superior colliculus and the LGN, which in turn project to the visual cortex (Grillo and Koulen, 2015).

The LGN is one of the most prominent nuclei in the primary visual pathway, acting as a relay station for retinal axons to transmit information from the visual bundle to light radiation, and then to the visual cortex (Xu et al., 2001; Dougherty et al., 2019). For primates, the LGN consists of six different layers, and these layers are made up of magnocellular cells (M cells) in the ventral layers (layers 1 and 2) and parvocellular cells (P cells) in the dorsal layers (layers 3–6). Each layer of M and P cells is separated by a layer of Koniocellular cells (K-cells). LGN layers 1, 4, and 6 receive input from the contralateral eye, and LGN layers 2, 3, and 5 receive input from the ipsilateral eye. After the axons of the RGCs reach the optic chiasm, the axons from the nasal halves of the two retinas pass through the optic chiasm and go into the contralateral optic tract, while the axons from the temporal halves go into the ipsilateral optic tract (Weyand, 2016; Ghodrati et al., 2017). For example, in the left LGN, the afferent retinal layer from the nasal hemiretina projects to the 1, 4, and 6 layers of the LGN, while the afferent retinal layer from the temporal hemiretina goes to the 2, 3, and 5 layers (see Figure 2). The largest category of the LGN thalamocortical cells is formed by the P cells, accounting for about 80% of the total, and M and K cells each contribute about 10% (Callaway, 2005). The three cell types of LGN inherit their properties from different types of RGCs (Schiller, 2010). P cells receive inputs from midget RGCs, and M cells receive input from parasol RGCs. The input of K cells to the retina is diverse, but many seem to come from small bistratified RGCs (Michael, 1988; Ghodrati et al., 2017). At present, damage to M cells, P cells, and K cells has been confirmed to occur during glaucoma in experimental animal models and human patients.

#### Magnocellular and Parvocellular Pathway Degeneration

Magnocellular (M) cells are located in inner two layers (layers 1 and 2) of the LGN. They function in the transmission of motor information, and are sensitive to high-frequencies. Parvocellular (P) cells make up the dorsal four layers (layers 3–6) of the LGN. They are sensitive to high-frequency space and transmit red and green color information (Schiller and Malpeli, 1978; Hendry and Calkins, 1998; Masri et al., 2020). Several studies have shown that both the M and P layers of the LGN are damaged in monkey glaucoma models (Dandona et al., 1991; Chaturvedi et al., 1993; Vickers et al., 1997; Weber et al., 2000; Yucel et al., 2000, 2001; Sasaoka et al., 2008; Ito et al., 2009, 2011; Yan et al., 2017), and this conclusion has also been confirmed in human glaucoma patients (Gupta et al., 2006, 2009; Dai et al., 2011; Hernowo et al., 2011; Shimazawa et al., 2012; Chen et al., 2013b; Lee et al., 2014; Wang et al., 2015, 2016; Zhang et al., 2016; Ersoz et al., 2017; Song et al., 2018; Xu et al., 2018; Li et al., 2019a; Kosior-Jarecka et al., 2020). Weber et al. used a laser-induced chronic glaucoma monkey model to explore changes in size, density, and number of LGN neurons during glaucoma. After histological staining, it was found that elevated intraocular pressure (IOP) significantly reduced the cross-sectional area of the LGN, and that the loss of cells in the M layers was about four times higher than the loss of cells in the P layers (38 vs. 10%). These results suggest that elevated IOP has a more severe effect on the degeneration of M layers than on the degeneration of P layers (Weber et al., 2000). However, in another study using the same model, when cytochrome oxidase (CO) histochemistry was used to analyze the functional afferents of surviving ganglion cells to the LGN and the primary visual cortex, no difference was found in the degree of decreased CO reactivity (COR) between neurons in the P and M layers of the LGN (Crawford et al., 2000). However, while it has been confirmed that both M and P layers of the LGN may be damaged during glaucoma, it is controversial whether one of these pathways is affected more than the other.

To further investigate whether there is a priority pathway in the atrophy of the LGN, another study in the same monkey glaucoma model used immunocytochemistry with antibody to parvalbumin to specifically label relay neurons connecting to the visual cortex. This study found that, in the glaucoma treatment group, the average cross-sectional area of relay neurons in M cell layer 1 and P cell layers 4 and 6 decreased by 28, 37, and 45%, respectively, compared with the control group (Yucel et al., 2001). Both this and previous results indicate that the relay neurons projecting from the LGN to the visual cortex experience significant atrophy during glaucoma, but this study also showed that neurons in the P cell layer experienced significantly greater atrophy than those in the M cell layer. In previous studies, it was also mentioned that the main damage in the P cell layer is in the form of neuronal atrophy, while damage in the M cell layer is mainly due to cell loss. Additionally, atrophy and loss in the M cell layer occur earlier than damage to the P cell layer (Weber et al., 2000).

Similar results have been observed in other studies, which show that the atrophy of neurons in the P cell layer is more severe than in the M cell layer (Sasaoka et al., 2008), but another study has found contradictory results (Ito et al., 2009). There are several possible explanations for these inconsistent or contradictory results. First, it could be due to inconsistency in the duration of study, leading to different degrees of atrophy measured in the end. It may also be due to different criteria used by different studies to identify neurons, that is, different nucleolus inconsistencies, which could bias some studies toward selecting neurons with less atrophy for area measurement. Finally, the staining method used in the monkey glaucoma model studies cited above labeled all the neurons in the LGN, while other studies focused only on the relay neurons in the LGN. This, combined with the fact that there are more intermediate neurons in the M cell layer than in the P cell layer, could also explain why the results are inconsistent. Injury to the LGN is a complex process, and the existing results show that there is atrophy and loss in both the large and small cell layer of the LGN, and that damage to the large cell layer occurs earlier than that to the small cell layer, but we cannot say whether either the large cell layer or the small cell layer suffers more damage. In addition to the glaucoma monkey model, researchers have constructed an experimental ferret model of high intraocular pressure to analyze the central damage of glaucoma (Fujishiro et al., 2014). In this model, Fujishiro et al. also found damage to the LGN, but, again, there was no priority pathway in terms of one cell type experiencing more damage than the other (Fujishiro et al., 2020).

#### Konio Cellular Pathway Changes in Glaucoma

Konio cells are located between the M and P cell layers of the LGN. K cells in different LGN layers have different characteristics of space, time, and contrast, so K cells are considered to be composed of many categories. Three subtypes of K cells can be identified by double labeling immunocytochemistry. One is a subclass containing only calbindin-D28k, the second is a subclass containing only the alpha form of calcium—calmodulin-dependent kinase II (αCaMK II), and the third subclass contains both of these proteins (Xu et al., 2001). The proportion of each type of cells differs between different K layers. This immune characteristic can be used to study damage to the K layer in LGN and visual cortex models of glaucoma.

In the glaucoma monkey model, the researchers used a CaMKII labeling method to detect K cells in the LGN. The results showed that the expression of CaMKII in the glaucoma treatment group was significantly lower than that in the control group. Unlike M and P cells, there is no significant linear correlation between the decrease in CaMKII and mean intraocular pressure, which may be related to the subtypes of K cells mentioned above (Yucel et al., 2003). At the same time, the researchers believe that damage to K cells occurs earlier than other types of cells. The sensitivity of the three types of cells to transneuronal degeneration may be related to anatomical factors, or it may be due to the different sensitivities of the cells themselves to IOP (Hendry and Reid, 2000; Xu et al., 2001; Yucel et al., 2001). Because of the diffusion structure of K cells between the layers of primate LGN, there are few studies on this kind of cells. However, in the ferret glaucoma model, K cells are projected into a separate layer in the LGN, just like M and P cells. In the ferret model, the cell types corresponding to M cells, P cells, and K cells are called X, Y, and W cells. In primates, K cells project to the middle layer of the LGN, while W cells project to layer C, which is located independently on the dorsal side of the ferret LGN. In this study, the number of neurons in layers A, A1 and C of the LGN decreased in eyes presenting with ocular hypertension (OH), and this study also used immunohistochemical analysis to specifically label neurons, astrocytes and microglia in LGN. It was found that NeuN, glial fibrillary acidic protein (GFAP) and Iba-1 immunoreactivity increased was observed in the LGN layer receiving OH eye projection. In addition, the results also showed that the damage to layer C was greatest, suggesting that the W cells projected to layer C were more sensitive and vulnerable to OH-induced neuronal damage than the X and Y cells projected to An and A1 layers (Fujishiro et al., 2020). However, the mechanism behind the damage to the K cells of primates and humans with glaucoma, needs to be further explored.

#### Possible Mechanism of Degeneration of LGN

Several studies have found that Nitric Oxide (NO) is associated with LGN neuronal death in chronic glaucoma models with LGN atrophy. There is experimental evidence that the expression of neuronal nitric oxide synthase (nNOS) and nicotinamide adenine dinucleotide phosphate-diaphorase (NADPH-d) in the LGN of rats increased after 1 week of elevated intraocular pressure (Wang et al., 2000). Furthermore, a study found nitrotyrosine immunoreactivity in LGN of a monkey glaucoma model, but not in the control group. According to these findings, we believe that LGN damage in glaucoma patients may be mediated by peroxynitrite (Luthra et al., 2005), and we speculate that high intraocular pressure may lead to an excessive release of NO in the LGN, resulting in neuronal death. At the same time, the activation of glial cells was also observed in the LGN of early glaucoma. In an ocular hypertension (OHT) primate model, GFAP immunoreactivity appeared in the LGN, and the loss of metabolic activity was accompanied by enhancement of GFAP immunoreactivity. This suggests that astrocytes also play a role in the atrophy of LGN neurons (Lam et al., 2009). It is also believed that endoplasmic reticulum (ER) stress is involved in neuronal death in the LGN. Proteins related to ER stress, such as the phosphorylation of eukaryotic initiation factor 2a (p-eIF2a) and C/EBP-homologous protein (CHOP), were observed in an LGN glaucoma model (Ito et al., 2011). Recently, deposition of amyloid beta (Aβ) and Tau protein in the LGN was found in a model of chronic glaucoma in monkeys (Yan et al., 2017), which is consistent with the deposition of Aβ and Tau protein in the retina of glaucoma patients (Gupta et al., 2016). According to the above findings, we believe that there are many possible mechanisms involved in neuronal death in the LGN in addition to RGC death, but the specific mechanism has not yet been confirmed.

### The Primary Visual Cortex

The axons from the M, P, and K layers of the LGN formation output pathway terminates in the primary visual cortex of the occipital lobe along the optic radiation (Prasad and Galetta, 2011; De Moraes, 2013). The primary visual cortex is mainly divided into six layers (I–VI): from the surface of the soft membrane to the white matter below. The cortical layers receiving inputs from LGN relay neurons from layers M, P, and K are layers IV and II-III, in which layer IV is further subdivided into four sublayers: IVA, IVB, IVC α, and IVC β. The M and P relay neurons project directly to IVC α and IVC β, respectively, and the neurons in layer K project to the II-III layers of the cortex (see Figure 1) (Yucel et al., 2003; Callaway, 2005; Ghodrati et al., 2017). In the following, we will describe in detail the visual cortex damage that has been found in primate glaucoma models and human glaucoma diseases.

#### Damage of Primary Visual Cortex in Glaucoma

In a monkey model of glaucoma, Vickers et al. observed not only an atrophy and loss of M and P cells, but also a periodic decrease of CO staining in the IVC α and IVC β layers of the V1 region. This result was confirmed by another study (Yucel et al., 2003). Therefore, researchers believe that there is trans-neuronal degeneration in the animal model of glaucoma, which leads to damage to the visual cortex (Vickers et al., 1997). Neuronal degeneration in glaucoma may be mainly due to a decrease in neuronal activity in the retina-LGN- visual cortex pathway. Interestingly, there are far more neurons in the visual cortex than in the LGN and the retina. In the glaucoma model of high intraocular pressure, the loss of neurons in the visual cortex was also shown to be much greater than that in the LGN, and the input of P cells into IVC β decreased more significantly than M cells in IVC α (14 vs. 8%). This result is consistent with the conclusion that damage to the P cell layers in the LGN is more severe than damage to the M cell layers of the LGN (Crawford et al., 2000). Since then, in the same research model, Crawford et al. found that the COR in layers II and III of V1 region, which received input from K cells, also decreased significantly (Crawford et al., 2001). In a recent study, the volume of the primary visual cortex of a spontaneous normal tension glaucoma marmoset model was significantly lower than that of the control group, and the number of cells in the IV layer of marmoset glaucoma was significantly lower than that of the control group (Noro et al., 2019). This result provides further evidence that there is atrophy of the central visual system in live animal models of glaucoma.

Due to the rapid development of detection technology in recent years, we can now observe changes in the visual pathways of human glaucoma patients, and damage to the visual cortex. In a case report by Gupta et al., magnetic resonance imaging (MRI) revealed that the thickness of the cortical band of the visual cortex in glaucoma patients was significantly lower than that in normal subjects, and they believe that the loss of neurons was related to the loss of visual field in patients (Gupta et al., 2006). In a recent systematic review, which incorporated results from 2,381 patients from 1,906 studies, neurodegeneration was found both inside and outside of the visual system. surface-based Morphometry (SBM) and voxel- based Morphometry (VBM) showed a decrease in the thickness and volume of the visual cortex; functional Magnetic Resonance Imaging (fMRI) and magnetic resonance spectroscopy (MRS) found functional and metabolic abnormalities in the primary visual cortex; and the resting-state fMRI (rs-fMRI) study found a disconnection between the primary and advanced visual cortex and between the visual cortex and related visual areas in glaucoma patients (Nuzzi et al., 2018). In another study, both normal-tension glaucoma (NTG) and primary open-angle glaucoma (POAG) showed atrophy of the visual cortex compared with negative control (NC), but the atrophy of the visual cortex of POAG was more severe than that of NTG. Furthermore, this study showed that, while the diffuse structural and functional abnormalities of the entire brain during glaucoma are partially due to nerve damage and degeneration caused by elevated intraocular pressure, they are also partially independent of elevated intraocular pressure and subsequent retinal degeneration (Giorgio et al., 2018). Recent imaging evidence shows that in the imaging examination of glaucoma patients, the effective connectivity between the visual cortex and the entire brain is significantly reduced (Li et al., 2020), and the decrease in visual acuity in POAG patients is driven by a combination of local (i.e., in the eyes) and broader (i.e., brain) effects (Di Cio et al., 2020). Studies of both animal models and human glaucoma patients have shown that there is damage to the central nervous system during glaucoma. This further defines glaucoma as a condition involving the spread of neurodegeneration.

With regard to the mechanism behind visual cortex injury, there are many possible factors involved, in addition to the factors related to neuronal damage in the LGN mentioned above, blood perfusion and nutritional factors in the visual cortex of patients with glaucoma are also significantly decreased. Damage to the LGN and visual cortex in glaucoma may increase the susceptibility of surviving RGCs to persistent damage by reducing the nutritional support of the LGN, leading to the progression of glaucoma (Zhang et al., 2015). At the same time, redox imbalance can also damage the glaucoma cortex. In the visual cortex of a rat glaucoma model, the expression of both Nicotinamide adenine dinucleotide phosphate oxidase 4 (NOX4) and inducible nitric oxide synthase (iNOS) increased, while the expression of nuclear factor erythroid 2-related factor 2 (Nfr2) decreased, demonstrating that cell signaling pathways that regulate antioxidant capacity are damaged during glaucoma (Hvozda Arana et al., 2020). Recent studies have also found that in the chronic glaucoma rat model, compared with the control group, glaucoma decreased mitochondrial ATP production, and the production of superoxide and hydrogen peroxide is increased (Hvozda Arana et al., 2021). These results indicate that glaucoma leads to impairment of mitochondrial function in the visual target area of the brain, accompanied by changes in the defense system of mitochondria and cytoplasmic enzymes. Redox imbalance can cause oxidative damage to macromolecules, which in turn affects the important functions of cells.

#### The Relationship Between Visual Cortex Activity and the Severity of Glaucoma and Visual Field Loss

It has been established that damage to any part of the visual pathway will lead to changes in the visual field, but the corresponding visual field changes do not occur as soon as the injury occurs. A study of visual cortex injury and visual field defects found that visual cortex activity began to decrease before patients experienced a loss of visual field (Murphy et al., 2016). Even when visual cortex activity begins to decrease, visual field performance remains at a stable level, and only when visual cortex activity decreases beyond the critical point does visual field loss become detectable. This suggests that glaucoma progresses and worsens in the eyes and brain before a substantial visual field defect is clinically detected. However, it is worth noting that this does not mean that the threshold of glaucoma can be directly inferred from the determined critical point, which still needs to be further confirmed through clinical studies.

Studies have shown that, compared with the normal control group, the visual cortex cerebral blood flow (CAG)/functional connectivity strength (FCS) ratio of POAG patients is significantly lower, and the CAG/FCS ratio is negatively correlated with the stage of glaucoma, but positively correlated with the visual field defect in the tongue gyrus of POAG patients (Wang et al., 2021). In an animal model of acute glaucoma, researchers tested the cerebral cortex of rhesus monkeys and cats and found that the increase in IOP inhibited the ability of the visual cortex to record fine details, and this effect gradually increased toward the center (Li et al., 2019b; Yuan et al., 2020). In addition, studies have found that there is a connection between the thickness and mobility of the visual cortex and the severity of glaucoma and have evaluated the cortical response of glaucoma patients and healthy subjects to 8 Hz flicker stimulation by using a functional MRI experiment with inherent blood oxygen levels dependent (BOLD) on contrast. In patients with severe glaucoma, stimulation of the upper or lower visual field showed a decrease in BOLD activity in the retinal localization area of the visual cortex. Among them, the primary visual cortex showed a greater loss of disease-driven activity than higher visual areas. Finally, it has also been found that the decrease of visual cortex activity in patients with POAG is well-correlated with the thickness of retinal nerve fiber layer (RNFL) and the results of perimetry (Yu et al., 2015). To sum up, we believe that visual cortex activity is a helpful gauge of the pathology of POAG and can be used as a clinical indicator of disease severity.

## Conclusions and Future Directions

In this review, we discuss evidence indicating that RGC damage caused by various factors (other than increased IOP) can lead to secondary damage to the retina and central neurons. For example, we discuss evidence that there is damage to M and P layer neurons in both experimental animal models of glaucoma and in human glaucoma; however, more research is required to settle the questions of whether there is a dominant pathway in neuronal damage in the LGN, and whether damage is earlier or more severe for the M or P layers (Weber et al., 2000; Crawford et al., 2001; Yucel et al., 2001). In the visual cortex, we have also found a decrease in the thickness and activity of the cortex in the glaucoma model, and this injury occurs before the visual field defect (Murphy et al., 2016), which suggests that the early diagnosis of the visual cortex is of great significance in controlling clinical manifestations of glaucoma. According to the current research results, we speculate that the pathogenesis of glaucoma may involve anterograde and retrograde degeneration processes of the retina and the central visual cortex. However, after RGCs and their surrounding glial cells are stimulated, the means to transmit the signal to the visual cortex of the CNS, and subsequent production of pathological changes in the central visual cortex, have become one of the key problems which need to be solved in the future. In addition, increasingly accurate methods need to be taken to determine whether neuronal damage in the retina is caused by RGC degeneration.

Glaucoma damage is not limited to the eyes, but also involves the central visual pathway (Rapino et al., 2018). Pathological changes during glaucoma may be the result of the anterograde synaptic transmission of RGCs, or the result of retrograde mechanism induced by neurodegenerative diseases that mainly affect the CNS (Davis et al., 2016). In view of this, drug therapy targeting IOP may not be sufficient to control glaucoma, and the study of synaptic plasticity may be an important new research direction in the treatment of glaucoma. A recent study showed that transneuronal degeneration occurs in a transsynaptic manner at the macro level, but demyelination precedes the loss of axons at cellular level. Activated astrocytes and macrophages cause demyelination, and axonal damage occurs after the loss of myelin. Demyelination of astrocytes and macrophages may be an early treatment target for glaucoma (You et al., 2019). Myelin-related proteins (such as Nogo-A) are the main inhibitors of neuronal plasticity and can cause permanent nerve damage to the CNS. A mouse model of glaucoma found that the inactivation of Nogo-A significantly promoted vision recovery and plasticity (Mdzomba et al., 2018), demonstrating that neuronal plasticity is another important new target for future research into the treatment of glaucoma.

Overall, these findings suggest that a better understanding of the mechanism behind RGC loss can not only develop new targets for the treatment of glaucoma, but can also provide information for the development of treatments for other neurodegenerative diseases (and vice versa). The future of glaucoma treatment involves not only for the retina, but the entire visual pathway.

## Author Contributions

XX and DJ: conceptualization. MY: methodology and writing- original draft preparation. RR and ZZ: formal analysis and investigation. XX and DJ: writing-reviewing and editing. All authors contributed to the article and approved the submitted version.

## Conflict of Interest

The authors declare that the research was conducted in the absence of any commercial or financial relationships that could be construed as a potential conflict of interest. The handling editor declared a shared affiliation with the authors at time of review.
